# ELECTROGASTROGRAPHY IN PEDIATRIC GASTROPARESIS: A SYSTEMATIC REVIEW AND META-ANALYSIS

**DOI:** 10.1590/S0004-2803.24612025-015

**Published:** 2025-10-27

**Authors:** Juan Javier PERALTA-PALMEZANO, Diana Paola ESCOBAR-SERNA, Fernando Javier PERALTA-PALMEZANO, Nancy Rocio ACOSTA-MURILLO, Rafael GUERRERO-LOZANO

**Affiliations:** 1 Department of Pediatrics, Universidad Nacional de Colombia. Colombia.; 2 Department of Pediatrics, HOMI-Fundación Hospital Pediátrico la Misericordia. Colombia.; 3 Department of Gynecology and Obstetrics, Universidad de Antioquia. Colombia.; 4 Department of Physiological Sciences, Universidad Nacional de Colombia. Colombia.

**Keywords:** Electrogastrography, electromyography, gastroparesis, stomach diseases, gastric dysrhythmia, gastric electrical activity, Eletrogastrografia, eletromiografia, gastroparesia, doenças do estômago, disritmia gástrica, atividade elétrica gástrica

## Abstract

**Background:**

Gastroparesis is a delay in gastric emptying without mechanical obstruction, lacking a clear pathophysiological mechanism, but with multiple histological abnormalities, including loss of interstitial cells of Cajal, which may alter slow waves. Slow waves can be assessed by electrogastrography. **Objective**: This study aimed to determine the prevalence and range of abnormalities in gastric slow waves in children with gastroparesis.

**Methods:**

We conducted a systematic review and meta-analysis following the Preferred Reporting Items for Systematic Reviews and Meta-Analyses guidelines (PROSPERO: CRD42023435301). Searches were performed in MEDLINE (PubMed), Embase, LILACS, Web of Science, and the Cochrane Register of Controlled Trials, from inception to September 2023, without language or publication restrictions. We included studies using surface electrogastrography in children (6-18 years) with gastroparesis. Outcomes included the percentage of recording time in normogastria (2-4 cycles per minute), tachygastria, and bradygastria; dominant frequency; power ratio; post-stimulus power change; and dominant frequency instability coefficient. Risk of bias was assessed using the Joanna Briggs Institute tool. Meta-analyses were conducted using random-effects models when appropriate, and heterogeneity was explored via the I² statistic and prediction intervals. When pooling was not feasible, a narrative synthesis was provided.

**Results:**

A total of 3730 articles were reviewed, four articles were included, with a total of 70 patients and 15 controls. When compared to controls, gastroparetics had significantly less fasting normogastria (Standardized Mean Difference = -3.363 [95% confidence interval: -4.068 to -2.657]), significantly more fasting tachygastria (Standardized Mean Difference = 3.287 [95% confidence interval: 2.657 to 3.918]), and significantly less power ratio (Standardized Mean Difference = -4.067 [95% confidence interval: -4.791 to -3.343]).

**Conclusion:**

Children with gastroparesis during fasting had a lower percentage of normogastria and higher percentage of tachygastria. Children with gastroparesis showed less change in post-stimulus power, reflecting possible alterations in gastric contraction and/or distension.

## HIGHLIGHTS

• This study aimed to determine the prevalence and range of abnormalities in gastric slow waves in children with gastroparesis.

• Children with gastroparesis had significantly less fasting normogastria than controls.

• Children with gastroparesis had significantly more fasting tachygastria than controls.

• Children with gastroparesis had a significantly lower power ratio than controls.

## RESUMO

**Contexto:**

A gastroparesia é um atraso no esvaziamento gástrico sem obstrução mecânica, carecendo de um mecanismo fisiopatológico claramente definido, mas com múltiplas anormalidades histológicas, incluindo a perda das células intersticiais de Cajal, o que pode alterar as ondas lentas gástricas. As ondas lentas podem ser avaliadas por meio da eletrogastrografia.

**Objetivo:**

Este estudo teve como objetivo determinar a prevalência e o padrão das anormalidades nas ondas lentas gástricas em crianças com gastroparesia.

**Métodos:**

Foi realizada uma revisão sistemática e meta-análise seguindo as diretrizes PRISMA (PROSPERO: CRD42023435301). As buscas foram feitas nas bases MEDLINE (PubMed), Embase, LILACS, Web of Science e Cochrane Register of Controlled Trials, desde a origem até setembro de 2023, sem restrições de idioma ou tipo de publicação. Foram incluídos estudos com eletrogastrografia de superfície em crianças (6 a 18 anos) com gastroparesia. Os desfechos avaliados incluíram a porcentagem do tempo de registro em normogastria (2-4 ciclos por minuto), taquigastria e bradigastria; frequência dominante; razão de potência (power ratio); alteração de potência pós-estímulo; e coeficiente de instabilidade da frequência dominante. O risco de viés foi avaliado com a ferramenta do Instituto Joanna Briggs. As meta-análises foram conduzidas com modelos de efeitos aleatórios quando apropriado, e a heterogeneidade foi avaliada pelo índice I² e pelos intervalos de predição. Quando não foi possível agrupar os dados, foi realizada síntese narrativa.

**Resultados:**

Um total de 3.730 artigos foi revisado; quatro estudos foram incluídos, totalizando 70 pacientes e 15 controles. Em comparação com os controles, crianças com gastroparesia apresentaram significativamente menos normogastria em jejum (diferença média padronizada = -3,363 [intervalo de confiança de 95%: -4,068 a -2,657]), mais taquigastria em jejum (diferença média padronizada = 3,287 [intervalo de confiança de 95%: 2,657 a 3,918]) e menor razão de potência (diferença média padronizada = -4,067 [intervalo de confiança de 95%: -4,791 a -3,343]).

**Conclusão:**

Crianças com gastroparesia em jejum apresentam menor percentual de normogastria e maior percentual de taquigastria. Além disso, demonstram menor variação de potência pós-estímulo, o que reflete possíveis alterações na contração e/ou distensão gástrica.

## INTRODUCTION

Gastroparesis is defined as a delay in gastric emptying without any evidence of mechanical obstruction. It affects the quality of life of patients and represents a significant economic burden on the health system^1^. Histological abnormalities in gastroparesis are heterogeneous and present in 83% of patients, including myenteric inflammation, decreased innervation, and structural changes in the gastric wall^2^ ^,^ ^3^. Inflammatory changes described are an abnormal immune infiltrate with increased macrophages, lymphocyte infiltrates, increased serum tumor necrosis factor α, and serum interleukin-6^2^ ^-^ ^6^. The structural changes in the gastric wall include fibrosis of the circular and longitudinal smooth muscle and loss of interstitial cells of Cajal (ICC), which is associated with delayed gastric emptying^2^ ^-^ ^7^. Innervation changes include decreased nerve fibers and ganglion cells^2^ ^-^ ^6^.

Electrogastrography (EGG) is the recording of gastric myoelectrical activity through electrodes that can be placed directly on the stomach serosa or abdominal wall, which evaluates the onset and propagation of gastric slow waves. This test may explain the pathophysiology of gastroparesis and allow objective evaluation of the response to treatment. Patients with gastroparesis exhibit abnormalities in the onset and conduction of slow waves^8^. Electrogastrogram abnormalities are associated with alterations in the number of ICC. In electrogastrographic records of patients with reduced ICC numbers, there were significantly fewer slow waves and more tachygastria when compared to the records of patients with an adequate ICC number, correlating significantly with an abnormal electrogastrogram^7^.

Gastroparesis is a poorly understood disease without a clear pathophysiological mechanism, and abnormalities in ICC have been observed. We conducted a systematic review and meta-analysis to evaluate gastric myoelectrical activity by assessing gastric slow waves using electrogastrography. Our aim was to determine the prevalence and range of abnormalities in gastric slow waves in children with gastroparesis using electrogastrography.

## METHODS

This systematic review was conducted in accordance with the Preferred Reporting Items for Systematic Reviews and Meta-Analyses (PRISMA) guidelines^9^. We followed the recommendations of the Cochrane Collaboration and Joanna Briggs Institute^10^ ^,^ ^11^. This study was registered in the International Prospective Registry of Systematic Reviews (PROSPERO, http://www.crd.york.ac.uk/PROSPERO) as CRD42023435301.

### Search Strategy

We conducted a systematic search of the Medline (PubMed), Embase, LILACS, Web of Science, and Cochrane Central Register of Controlled Trials databases, collecting publications from inception to September 2023. No restrictions on the language, publication format, or publication date were included in the search. The detailed search strategy is provided in SUPPLEMENTARY MATERIAL 1 . We screened the reference lists of all the review articles identified through our electronic search for additional studies. We checked the reference lists of all included primary studies for additional references. We contacted the corresponding authors of the published and ongoing trials to seek recent or additional data.

**SUPPLEMENTAL MATERIAL 1.**
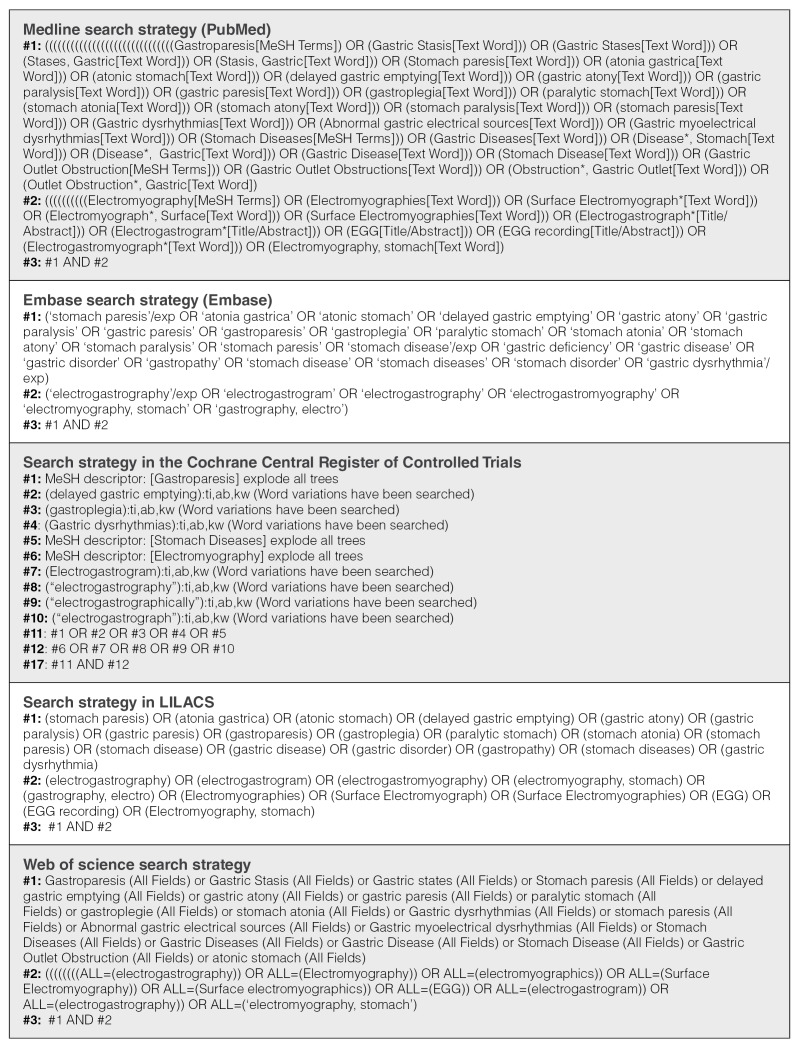
Search Strategies.

### Inclusion and exclusion criteria

We included original research articles in which electrogastrography was used to evaluate children with gastroparesis. We excluded review articles, letters/correspondence, short communications, editorials/opinions, commentaries, pictorial essays, gray literature such as conference proceedings, posters, abstracts, government reports, and online forums for reports from for-profit and non-profit organizations. 

We included studies with patients aged between 6 and 18 years who were diagnosed with gastroparesis and underwent electrogastrography. Gastroparesis was defined as a delay in gastric emptying in patients with upper gastrointestinal symptoms, without a mechanical obstruction^12^. If the term ‘gastroparesis’ was not explicitly mentioned, we included studies if the description of the population met all three criteria: (1) the presence of compatible symptoms, (2) objective evidence of delayed gastric emptying, and (3) absence of structural gastrointestinal pathology^13^. Studies that included only healthy subjects or those with poorly defined disease were excluded. When the full text was not available, the authors were contacted to obtain this information. We also included pretreatment electrogastrographic recordings from studies that investigated the treatment of patients with gastroparesis. We excluded studies in which invasive electrogastrographic (i.e., serous or mucosal) recordings and high-resolution techniques were performed, because they represent different methodologies that are not comparable to surface electrogastrograms. We excluded studies in which there was surgical manipulation or alteration of the gastrointestinal anatomy prior to the electrogastrogram measurement.

### Study selection and data extraction and management

Two authors independently screened titles and abstracts for inclusion and subsequently excluded non-relevant ones. A random sample of 10% of the titles was checked to ensure an accurate capture. Discrepancies were discussed between the authors, with a third reviewer mediation if necessary. The selected results were then screened for full text. A list of articles was generated for full-text review and data were independently extracted by two authors using a form developed by the authors. Discrepancies were resolved by consensus through the participation of a third author. Attempts were made to contact the corresponding author by e-mail if the data were not available. We extracted information regarding the author, year of publication, study title, study site (country), study design, diagnostic criteria used, population, inclusion and exclusion criteria, and reference ranges. Data from the electrogastrography methodology, such as the number and type of electrodes, skin preparation, and electrode placement method. We also extracted pre-specified primary and secondary outcomes. The extracted data were recorded in Microsoft Excel (Microsoft 365, version 2311).

### Risk of bias assessment

Two authors independently assessed the risk of bias using the risk of bias assessment tool described in the Joanna Briggs Institute Evidence Synthesis Handbook^10^. Disagreements were resolved through discussion and review by a third reviewer. We assessed the risk of bias of the outcomes specified for inclusion in the outcome summary tables based on the following questions: (1) Was the sampling frame appropriate for addressing the target population? (2) Were study participants appropriately recruited? (3) Was the sample size sufficient? (4) Were the study participants and settings described in detail? (5) Was data analysis performed with sufficient coverage of the identified sample? (6) Were valid methods used to identify the condition? (7) Was the condition measured in a standard and reliable manner for all the participants? (8) Was the statistical analysis appropriate? (9) Was the response rate adequate? Otherwise, the low response rate was adequately managed.

Each potential source of bias was rated as yes, no, unclear, or not applicable, based on the criteria outlined in the Joanna Briggs Institute’s Evidence Synthesis Handbook^10^. The tool does not allow for a summary assessment of the risk of bias; therefore, we conducted only a narrative review of the results. In cases of uncertainty regarding the methodology or data, we contacted the study authors for clarification. 

### Outcomes

Clinical criteria used to diagnose gastroparesis were recorded in each article along with any additional eligibility criteria used in each study. Primary and secondary outcomes were recorded under both fasting and post-stimulus conditions as described in previous studies. The primary outcome measured was the percentage of the recording duration during which the dominant power was within the frequency ranges of normogastria, bradygastria, and tachygastria. In humans, the normal percentage of slow gastric waves is defined as ≥70%^14^.

The secondary outcomes measured were dominant frequency (DF), power ratio (PR), change in post-stimulus power (IDP), dominant frequency instability coefficient (DFIC), and the relationship between electrogastrogram results and symptoms reported by study subjects. We also extracted data from studies that assessed the relationship between electrogastrographic parameters and histological findings from gastric biopsies. When reported, we recorded statistical associations between EGG measurements (e.g., rhythm patterns, dominant frequency, power ratio) and biopsy markers, such as inflammatory cell density or structural changes. These outcomes were included only if directly analyzed and reported by the original authors. Measurements of the absolute value of amplitude or power were not analyzed because they are influenced by many factors, such as abdominal wall thickness, skin preparation and conductance, and electrode position^15^. Instead, measures such as power ratio and post-stimulus power increase were analyzed, as these postprandial changes better reflect changes in gastric muscle activity^16^. The power ratio was defined as the ratio of power recorded before and after the intervention, wherein a result >1 reflected an increase in gastric contractility due to the intervention, whereas <1 reflected a decrease in gastric contractility. Changes in post-stimulus power were defined as the difference between the power of the baseline and post-intervention recordings, a measurement that is applied when measuring power in decibels. The dominant frequency instability coefficient was defined as the ratio of the standard deviation (SD) to the mean (M) of the DF and power of the electrogastrogram (DFIC=SD/M × 100%)^14^.

### Statistical analysis

Statistical analyses were performed using R (version 4.3.1; 2023 The R Foundation for Statistical Computing, Vienna, Austria). We used the meta and metafor packages and the metoprop, metocont, and metanean functions. If statistical pooling was not appropriate, a narrative summary was performed using the ‘summary of effect estimates’ method, as described in the Cochrane Handbook^11^.

Meta-analyses were performed when there were sufficient similarities between studies with respect to intervention and outcome measures. If the data were presented as medians and interquartile ranges or ranges, the means and standard deviations were calculated using the formulas described by Wan et al.^17^. or Hozo et al.^18^. Data were presented as frequency (n) and percentage (%) for categorical outcomes and as mean ± standard deviation for continuous outcomes. Here, we report the mode for the number of electrogram electrodes used.

Where controlled studies were available, the measured electrogastrogram results were combined using a random-effects model with the DerSimonian-Laird estimator for inter-study variance with a 95% confidence interval (CI)^19^. A random-effects model was chosen because of the expected heterogeneity between studies owing to variability in experimental methods. Because different methodologies are used in the electrogastrogram technique for continuous data, we estimated the standardized mean difference (SMD) based on Hedges’ g (adjusted) for comparison between patients and controls because of the small sample sizes in many of the included studies^20^. There was no difference observed between the groups when the SMD was zero. If the SMD result was negative, the mean electrogastrogram measurements in the patient group were lower than those in the control group. For effect size, we considered a small effect for an SMD less than 0.4, a moderate effect between 0.4-0.7, and a large effect if it was greater than 0.7^11^ ^,^ ^21^. We also reported the SMD 95%CI; if the CI encompassed zero, it was considered not statistically significant^22^.

For the meta-analysis of proportions, to address the asymmetry in the distribution of the observed proportions, we applied transformations, thus improving the validity of subsequent statistical analyses^23^. During which the transformed proportions and their inverted variances (i.e., study weights) were used for all calculations. The summarized ratio and its confidence interval were then converted back to the original proportion metric for easy interpretation^24^. As small sample sizes were expected and/or extreme proportions needed to be addressed, we used the Freeman-Tukey double-arc transformation^25^.

Heterogeneity was measured using the I^2^ test, and considerable heterogeneity was considered if the I^2^ was ≥75%^11^. We also measured the prediction interval (PI), presenting heterogeneity on the same scale as the original results and not in proportions^26^. In homogeneity, the PI matches the summarized effect CI, and in heterogeneity, the PI covers a wider range than the CI^27^. We evaluated the bias of small studies using R, meta and metafor packages and funnel and metabias functions. For meta-analyses comparing measurements in electrogastrogram recordings between gastroparetic patients and controls, we first assessed the biases of small studies using a visual aid; therefore, we performed funnel plots. If the number of studies in the meta-analysis allowed it (k>10), the evaluation was complemented using Egger’s test. Publication bias was assessed using the Vevea and Hedges weight function model, using the WeightR package and WeightFunct function*.* In this model, we performed a likelihood ratio test, with a *P*-value <0.1 indicating a publication bias^28^.

## RESULTS

**FIGURE 1.**
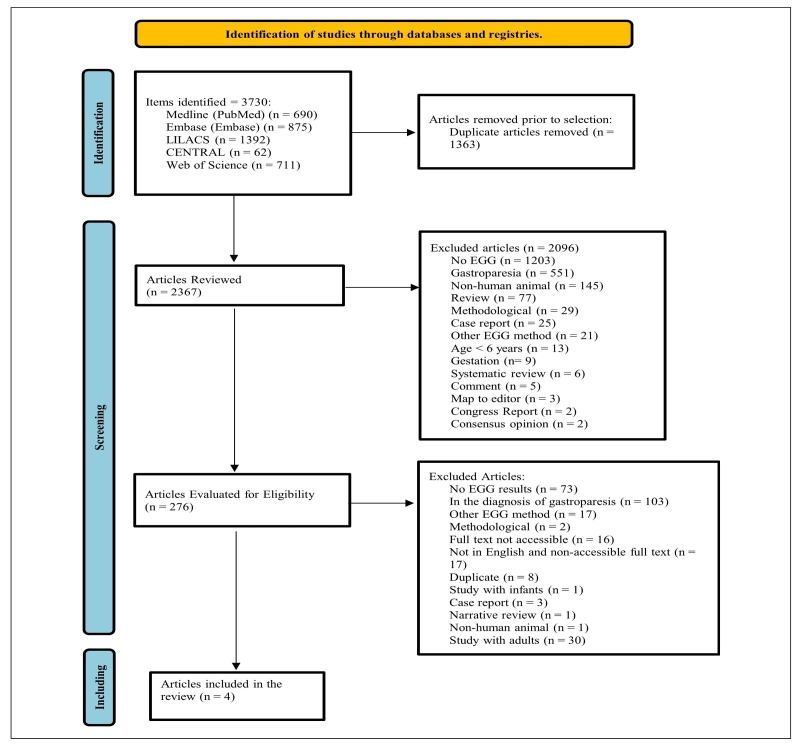
Preferred reporting items for systematic reviews and meta-analyses (PRISMA) flowchart of the review process.

Our database search identified 3730 articles, of which four were included. Figure 1 shows the flowchart of the review process. Table 1 presents the characteristics of the included studies^29^ ^-^ ^32^. Four studies included 70 patients and 15 controls with EGG records. The sex distribution was similar in children with gastroparesis (51.5% women versus 48.5% men). Sex was not reported in two studies^30^ ^,^ ^32^. Only one study performed EGG in control children, with more women (57.1% women versus 42.9% men). Of the included studies, two (50%) were conducted in the United States^29^ ^,^ ^32^ and two (50%) in Italy^30^ ^,^ ^31^. All studies were prospective. Two (50%) studies included patients with diabetic gastroparesis^30^ ^,^ ^31^ and two (50%) patients with functional dyspepsia with delayed gastric emptying^29^ ^,^ ^32^. Among the included studies, two used gastric emptying scintigraphy to confirm delayed gastric motility^29^ ^,^ ^32^, and two used gastric antral ultrasonography^30^ ^,^ ^31^. Reported symptoms included nausea, vomiting, early satiety, abdominal pain, postprandial fullness, and anorexia. While no study applied a standardized clinical scoring system, all described symptoms compatible with gastroparesis or functional dyspepsia. In two studies^31^ ^,^ ^32^, mechanical obstruction and peptic ulcer disease were explicitly excluded through imaging or endoscopy. SUPPLEMENTARY TABLE 1 shows additional clinical definitions and study characteristics**.**

**TABLE 1.** Characteristics of the included studies.

Study	Year	Design	Country	Patients n (% men)	Patients age (years)	Controls n (% men)	Controls age (years)	Disease
Barbar et al.^29^	2000	Prospective, non-randomized, uncontrolled study	United States	5 (40)	12.4			Functional dyspepsia + delayed gastric emptying
Cucchiara et al.^30^	1998	Prospective, non-randomized, controlled study	Italy	26 (NR)	9	15 (40)	7	Diabetic gastroparesis
Franzese et al.^31^	2002	Prospective, non-randomized, controlled study	Italy	28 (50)	11.8			Diabetic gastroparesis
Friesen et al.^32^	2005	Prospective, non-randomized, uncontrolled study	United States	11 (NR)	NR			Functional dyspepsia + delayed gastric emptying

*NR: not reported.

**SUPPLEMENTARY TABLE 1:** . Pediatric studies on delayed gastric emptying: definitions, criteria, and histology.

Reference	Disease evaluated	How was delayed gastric emptying defined?	Inclusion criteria	Exclusion criteria	Were gastric histological studies performed? Evaluation of CCIs (yes / no / do not describe)
Barbar et al. 2000	Functional dyspepsia + delayed gastric emptying	Gastric emptying scintigraphy test: The half-life (T1/2) of gastric emptying was calculated. Normal gastric emptying was defined as T1/2 in a range of 60 to 90 minutes, and delayed emptying was defined as T1/2 of more than 90 minutes.	Children scheduled for solid gastric emptying study. With gastrointestinal symptoms, each patient had at least one of the following symptoms: nausea, vomiting, abdominal pain, or early satiety. With delayed gastric emptying. They do not specify whether they ruled out a mechanical obstruction	Not reported	No
Cucchiara et al. 1998	Diabetic Gastroparesis	Ultrasonography of the gastric antrum: The stomach was considered empty when the area of the antral section returned to baseline and persisted unchanged for at least 30 minutes and no food particles were observed in the antral lumen.	Insulin-dependent diabetes mellitus in paediatric patients with dyspeptic symptoms: abdominal pain (epi and mesogastric), early satiety or anorexia, feeling of abdominal fullness (or bloating), regurgitation (or vomiting or heartburn). Delayed gastric emptying	Patients taking medications known to interfere with gastrointestinal motility were not included.	No
Franzese et al. 2002	Diabetic Gastroparesis	Ultrasonography of the gastric antrum: The stomach was considered empty when the area of the antral section returned to baseline and persisted unchanged for at least 30 minutes and no food particles were observed in the antral lumen. Gastric emptying time (minutes) is expressed as mean values and ranges	Children with insulin-dependent diabetes mellitus + recurrent symptoms of upper gastrointestinal dysfunction + No patients had received antisecretory or prokinetic drugs for at least 12 weeks prior to the study + Studies with barium and/or upper endoscopy had excluded mechanical obstruction and peptic ulcer disease of the gastrointestinal tract, respectively in all patients + Delayed gastric emptying determined by antrum ultrasonography gastric.	Not reported	No
Friesen et al. 2005	Functional dyspepsia + delayed gastric emptying	Gastric Emptying Scintigraphy Test: Delayed gastric emptying was defined as gastric retention >70% at one hour or >50% at 2 hours.	Dyspepsia was defined as chronic or recurrent pain or discomfort in the upper abdomen, consistent with published criteria for the diagnosis of functional dyspepsia + All patients had no gross pathology (including nodularity, erosion, and ulceration) on endoscopic examination and all had the presence of *H. pylori*. excluded by histology and rapid urease test on antral biopsies (it is not specified whether they ruled out a mechanical obstruction). Delayed gastric emptying	Not reported	CD25, CD3, CD20, and tryptase-positive lamina propria cells were listed by counting cells within a 1 × 1 mm grid in 5 high-power fields. The mean value obtained was expressed as the number of cells per square millimeter.

### Percentage of dominant power in electrogastrography measurements

**Figure 2.**
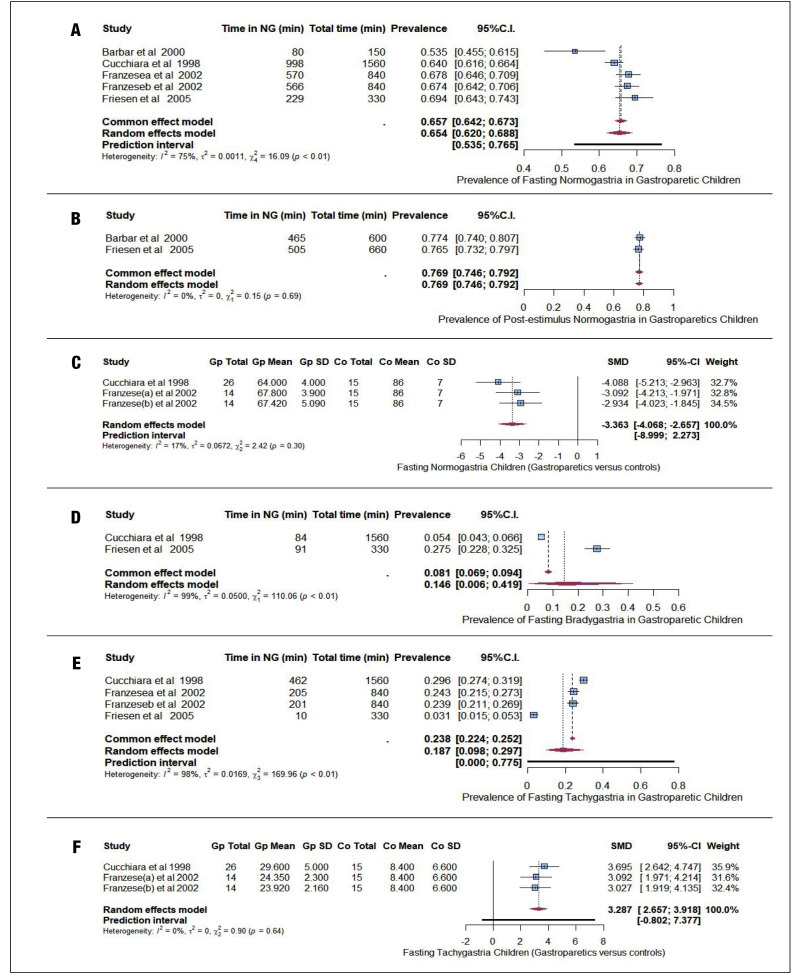
A) Pooled prevalence and 95% confidence intervals for fasting normogastria in children with gastroparesis. B) Pooled prevalence and 95% confidence intervals for post-stimulus normogastria in children with gastroparesis. C) Pooled standardized mean difference and 95% confidence intervals for prevalence of fasting normogastria in children. D) Pooled prevalence and 95% confidence intervals for fasting bradygastria in children with gastroparesis. E) Pooled prevalence and 95% confidence intervals for fasting tachygastria in children with gastroparesis. F) Pooled standardized mean difference and 95% confidence intervals for prevalence of fasting tachygastria in children.

Of the included studies, four reported information on the percentage of fasting normogastria in children with gastroparesis. In the pooled data of the four studies^29^ ^-^ ^32^, there was a percentage of 65.4% (95%CI: 62-68.8%, I^2^=75%, PI: 53.5-76.5%) (Figures 2A to F). Two studies presented information on the percentage of post-stimulus normogastria in children with gastroparesis. In the pooled data from the two studies,^29^ ^,^ ^32^ there was a percentage of 76.9% (95%CI: 74.6 to 79.2%, I^2^=0%) (Figure 2B). One study reported information on the percentage of fasting normogastria in controls,^30^ with a percentage of 86%±7 (mean ± standard deviation). No studies reported the percentage of post-stimulus normogastria in controls. We found two controlled studies that reported information on the percentage of fasting normogastria^30^ ^,^ ^31^. The pooled data from these studies showed a lower percentage of fasting normogastria in gastroparetics, with a significant difference and a large effect (SMD = -3.363 [95%CI: -4.068 to -2.657], I^2^ = 17%, PI: -8.999 to 2.273) (Figure 2C). No studies reported the percentage of post-stimulus normogastria in controls.

Of the included studies, two reported information on the percentage of fasting bradygastria in gastroparetics. In the pooled data from these two studies,^30^ ^,^ ^32^ there was a percentage of 14.6% (95%CI: 0.6-41.9%, I^2^=99%) (Figure 2D). One study presented results on the percentage of post-stimulus bradygastria in gastroparetics, with a percentage of 15.9%±13% (mean ± standard deviation)^32^. One study reported information on the percentage of fasting bradygastria in controls, with a percentage of 3.8%±2.7% (mean ± standard deviations)^30^. Only the study by Cucchiara et al.^30^ reported data comparing gastroparetic patients with controls, with no significant difference in the percentage of fasting bradygastria (5.4%±4% versus 3.8%±2.7%, respectively; NS [mean ± standard deviation]). No pediatric studies compared post-stimulus bradygastria between patients with gastroparesis and healthy controls.

Of the included studies, three reported information on the percentage of fasting tachygastria in gastroparetics^30^ ^-^ ^32^. In the pooled data from these studies, there was a percentage of 18.7% (95%CI: 9.8-29.7%, I^2^=98%, PI: 0-77.5%) (Figure 2E). One study presented information on the percentage of post-stimulus tachygastria in gastroparetics, with a percentage of 3.1%±5.1 (mean ± standard deviation)^32^. One study reported information on the percentage of fasting tachygastria in controls, with a percentage of 8.4%±6.6 (mean **±** standard deviation)^30^. No study has reported the percentage of post-stimulus tachygastria in controls. We found 2 controlled studies that reported information on the percentage of fasting tachygastria^30^ ^,^ ^31^. The pooled data from these studies showed a higher percentage of fasting tachygastria in gastroparetic patients, with a significant difference and a large effect (SMD=3.287 [95%CI: 2.657-3.918], I^2^=0%, PI: -0.802 to 7.377) (Figure 2F). No study has reported the percentage of post-stimulus tachygastria in controls.

### Dominant frequency

None of the included studies reported fasting or post-stimulus dominant frequency.

### Dominant frequency instability coefficient

Barbar et al. reported DFIC in children with gastroparesis, with a mean of 0.256 for fasting recordings and 0.186 for post-stimulus recordings; however^29^. None of the included studies reported DFIC in controls.

### Change of post-stimulus dominant power

Friesen et al. reported changes in post-stimulus dominant power in children with gastroparesis, with 2.9 dB±4.2 dB (mean ± standard deviation)^32^. None of the included studies reported changes in the post-stimulus dominant power in control children.

### Power ratio

**Figure 3.**
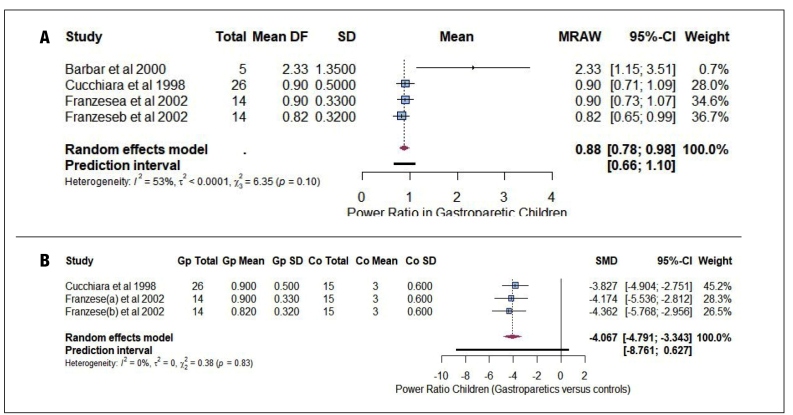
A) Pooled mean raw and 95% confidence intervals for power ratio in gastroparetic children. B) Pooled standardized mean difference and 95% confidence intervals for power ratio in children.

Three studies presented information on power ratio in gastroparetic children^29^ ^-^ ^31^. Pooled data from these studies summarized a mean of 0.88 (95%CI: 0.78 to 0.98, I^2^=53%, PI: 0.66 to 1.10) (Figures 3A and B). Cucchiara et al. reports a power ratio in control children of 3.0±0.6 (mean ± standard deviation)^30^. Of the controlled studies with electrogastrogram recordings, we included two that reported power ratio data in children. Franzese et al. reported two groups of patients that were analyzed separately. In the combined data from these two studies, there was a lower power ratio in gastroparetic children, with significant difference and large effect (SMD= -4.067 [95%CI: -4.791 to -3.343], I^2^=0%, PI: -8.761 to 0.627) (Figure 3B)^30^ ^,^ ^31^.

### Relationship of symptoms with electrogastrography

Friesen et al. found no significant differences in symptom frequency or symptom severity in patients with normal versus abnormal EGG^32^. Cucchiara et al. reported that patients with delayed gastric emptying showed a significantly lower fed-to-fasting power ratio (0.9±0.5) than those with normal gastric emptying time (2.8±0.8, *P*<0.01) and control subjects (3.0±0.6, *P*<0.01), with a significant inverse correlation between fed-to-fasting power ratio and gastric emptying time in both patients and control subjects (r=-0.91, *P*<0.01)^30^.

### Relationship of gastric biopsies with electrogastrography

Friesen et al. compared biopsy results with electrogastrogram records to evaluate inflammatory cells, and found that both mean and maximum CD3+ cell densities were significantly correlated with the percentage of normal slow waves (r=0.486, *P*=0.02, and r=0.500, *P*<0.02, respectively). There was a significant negative correlation between the mean and maximum CD3+ densities and bradygastria percentage (r=-0.479, *P*=0.02; and r=-0.509, *P*<0.02, respectively). CD3+ mean and peak densities were significantly increased in patients with a normal percentage slow wave in the pre-prandial state (27.4 vs 15.5, *P*=0.009, and 35.4 vs 19.4, *P*=0.003, respectively) but not in the postprandial state^32^.

### Electrogastrography methodology

The four included studies used three electrodes, using silver-silver chloride electrodes to register myoelectrical activity with bipolar recordings. Four studies reported the method of skin preparation prior to electrode placement, indicating that the skin was cleaned and abrased with paste. Only one study reported that the skin was shaved if hair was present prior to electrode placement to improve the conductance^32^. Three studies (75%) used ultrasonography to guide electrode placement^29^ ^-^ ^31^, and one (25%) used anatomical landmarks (xiphoid process, umbilicus, and left costal border with the midclavicular line) to determine the electrode position^32^.

One study (25%) did not specify whether medications were discontinued prior to the electrogastrogram^32^, two studies (50%) reported that subjects were not taking any medications for at least 12 weeks prior to the test^30^ ^,^ ^31^, and one study (25%) discontinued medications that affected gastrointestinal motility three days prior to the study^29^. One study (25%) had at least a 6-hour fast prior to the study^29^, three (75%) had overnight fasts prior to the test^30^ ^-^ ^32^. Four studies reported the use of meal stimuli using solid and liquid foods, with variations in food and caloric intake between studies.

The overall duration of the recording was reported in four studies, ranging from 90 to 150 minutes, with a mean duration of 120 minutes. The duration of fasting recording was reported in four studies, ranging from 30 to 60 minutes, with a mean duration of 45 minutes. The duration of post-stimulus recording was reported in four studies, ranging from 60 to 120 minutes, with a mean duration of 75 minutes. Gastric slow-wave frequencies defined as normogastria in the studies were on average 2.1 cpm (range: 2-2.4 cpm) and 3.9 cpm (range: 3.7-4 cpm) for the lower and upper limit, respectively. A more detailed description of the methodology used in the studies is presented in SUPPLEMENTARY TABLE 2.

**SUPPLEMENTARY TABLE 2.** Electrogastrography protocols and parameters in pediatric studies of gastric motility.

Reference	Barbar et al. 2000	Cucchiara et al. 1998	Franzese et al. 2002	Friesen et al. 2005
Number of electrodes	3	3	3	3
Type of Electrodes	Ag-Ag pregelatinized electrocardiogram electrodes	Ag-AgCl	Ag-AgCl	Ag-AgCl
Type of Records	Bipolar	Bipolar	Bipolar	Bipolar
Preparing the skin before placing the electrodes	Light abrasion of the skin with a sandy skin preparation paste, followed by the application of an “electro” cream.	The epigastric skin previously cleaned and abrased with a paste (OmniPrep, D.O. Weaver, Aurora, CO).	The epigastric skin previously cleaned and abrased with a paste (OmniPrep, D.O. Weaver, Aurora, CO).	The epigastric skin where electrode swere to be positioned was shaved, as necessary to remove any hair, cleaned, and abraded with sandy skin preparation jelly (OmmiPrep; Weaver, Aurora, CO) to reduce the impedance.
Electrode Placement Metho d	Guided by gastric ultrasonography	Guided by gastric ultrasonography	Guided by gastric ultrasonography	Standard Method
Description of the Placement Method	Two epigastric electrodes were connected to produce a bipolar EGG signal and a third electrode was placed over the left flank region as a reference grounding electrode.	Electrodes were placed on the epigastric skin along the distal gastric longitudinal axis after ultrasound localization of the stomach: the first electrode was placed approximately at the midpoint between the umbilicus and the xiphoid; the second was placed on the left side of the subject, just below the lower rib and above the level of the first electrode; and a reference electrode was placed 4-6 cm to the right of the midline and 2 cm above the navel.	An electrode was located in the midline of the abdomen, several centimeters above the navel, after ultrasound localization of the antrum; The second electrode was located on the left side of the subject, just below the lower rib and above the level of the first electrode. A reference electrode was placed in the left iliac fossa.	Two silver-silver chloride (DNA, Dayton, OH) EGG electrodes were placed on the abdominal skin. An electrode was placed at the midpoint between the xiphoid process and the navel. The second electrode was placed in the sternal middle on the left side of the subject, just below the lower rib and above the level of the first electrode. A reference electrode was placed in the lower quadrant near the left costal margin.
Were the medications discontinued prior to EGG registration?	Medications that are known to affect gastrointestinal motility during the 3 days prior to the study.	They were not taking medications that interfere with gastrointestinal motility.	No patients had received antisecretory or prokinetic drugs for at least 12 weeks prior to the study.	NR
Duration of fasting before the study.	6 hours or more before the study.	Overnight	Overnight	Overnight
Stimulus used	Meal	Meal	Meal	Meal
Description of the stimulus	Two scrambled eggs mixed with technetium sulfur colloid with two pieces of toast and 4 oz orange juice	Mixed solid-liquid meal based on caloric intake at breakfast for children of different ages (bread + raw ham + butter + fruit juice)	Mixed solid-liquid meal based on caloric intake at breakfast for children of different ages (bread + raw ham + butter + fruit juice)	Two whole eggs labeled with sulphur-colloid 99mTc and 120 ml of water.
Total Recording Duration (minutes)	150	120	120	90
Fasting Recording Duration (minutes)	30	60	60	30
Post-stimulus recording duration (minutes)	120	60	60	60
Lower Normal Frequency Limit (cpm)	2.4	2	2	2
Upper Limit of Normal Frequency (cpm)	3.7	4	4	4
How was establish the normal frequency range?	Self-defined (no published reference standard is used)	Self-defined (no published reference standard is used)	Self-defined (no published reference standard is used)	Self-defined (no published reference standard is used)
Measurements and definitions of EGG parameters	(1) The dominant frequency (DF). (2) Percentage of DP in the normal frequency range (2.4 to 3.7 cpm), the bradygastric range (0.5 to 2.4 cpm), and the tachygastria range (3.7 to 9 cpm). Nine to 15 cpm were removed as an artifact and separated from tachygastria, because they were supposed to arise from the duodenum or lung. (3) Dominant frequency instability coefficient (DFIC), a measure of how much the DF changes over the recording period. (4) The postprandial and fasting potency ratio; The absolute value of power was not reported because it is influenced by many factors (such as the thickness of the patient’s abdominal wall, skin preparation and conductance, and the position of the electrodes).	(1) Percentage of time during which gastric arrhythmias are observed in the EGG; Arrhythmias included Bradygastrian (dominant peak was in the range of 0.5 to 2.0 cpm); tachygastria (the dominant peak was in the range of 4.0 to 9.0 cpm); An arrhythmic episode had to be recorded for at least 2 minutes with simultaneous absence of normal signal. (2) Postprandial-fasting potency ratio of dominant EGG (potency ratio).	(1) Percentage of normal gastric electrical rhythm. (2) Tachygastria percentage (percentage of time during which the spectrum has a dominant peak in the range of 4.0 to 9.0 cpm) (3) feed-to-fast ratio of the dominant power of the electrogastrogram (power ratio).	(1) Dominant frequency (frequency at which the power spectrum of an EGG recording had a maximum power in the range of 0.5 to 9 cpm). (2) Key power (the power at the key frequency in the power spectrum of the EGG register. (2) Postprandial EGG (δP) dominant potency change (The difference between the dominant potency of EGG after and before the test meal). (4) Percentage of normal slow waves and percentage of arrhythmias (the percentages of time during which regular slow waves of 2 to 4 cpm and electrical arrhythmias, respectively, were present during the entire observation period). An arrhythmic episode required a frequency of either >4 or <2 cpm and had to be recorded for ≥2 min with the normal signal simultaneously absent.

### Risk of bias quality assessment

**Figure 4.**
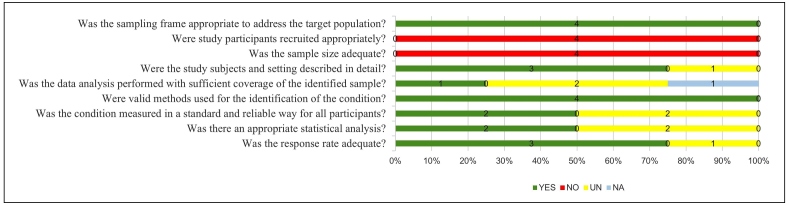
Study quality assessment summary chart with JBI critical assessment checklist for studies reporting prevalence data (answers: yes, no, un (unclear) or na (no/applicable).

The results of the quality assessment are shown in Figure 4. In the evaluation of the methodological quality of the included studies, it was evident that the sampling frame was adequate to address the target population in all included studies. Regarding participant recruitment, none of the studies employed an adequate recruitment strategy. The sample size was inadequate in all studies included because of the lack of evidence that the authors performed a sample size estimate, and the studies were small, with fewer than 88 patients. An adequate description of the subjects was provided in 75% of the studies. We assessed the coverage bias between the study subgroups using data analysis with sufficient coverage of the samples analyzed. There were no subgroups in 25% of the included studies; therefore, they were not applicable. Of the subgroup studies, 25% had data analysis with sufficient coverage, with an unclear coverage of 50%. All studies reported valid methods for identifying gastroparesis. The information provided regarding the measurement of the condition in 50% of the studies was unclear, considering that the training of those performing the tests or clinical or research experience was not clearly described, or whether there was more than one assessor, whether a comparison was made between the results, or whether the measurement was the same for all participants. Appropriate statistical analyses were performed in 50% of studies. The response rate was adequate in 75% of studies. The results of the quality assessment are presented in SUPPLEMENTARY TABLE 3.

**SUPPLEMENTARY TABLE 3.** Methodological quality appraisal of prevalence studies using the JBI checklist.

Reference	Barbar et al. 2000	Franzese et al. 2002	Friesen et al. 2005	Cucchiara et al. 1998
Was the sampling frame appropriate to address the target population?	YES	YES	YES	YES
Were study participants recruited appropriately?	NO	NO	NO	NO
Was the sample size adequate?	NO	NO	NO	NO
Were the study subjects and setting described in detail?	UN	YES	YES	YES
Was the data analysis performed with sufficient coverage of the identified sample?	UN	UN	NA	YES
Were valid methods used for the identification of the condition?	YES	YES	YES	YES
Was the condition measured in a standard and reliable way for all participants?	UN	UN	YES	UES
Was there an appropriate statistical analysis?	UN	YES	YES	UN
Was the response rate adequate? If not, was the low response rate adequately managed?	YES	UN	YES	YES

JBI critical appraisal checklist for studies reporting prevalence data (answers: yes, no, unclear or no/applicable).

### Small-study effect and publication bias

**SUPPLEMENTAL FIGURE 1.**
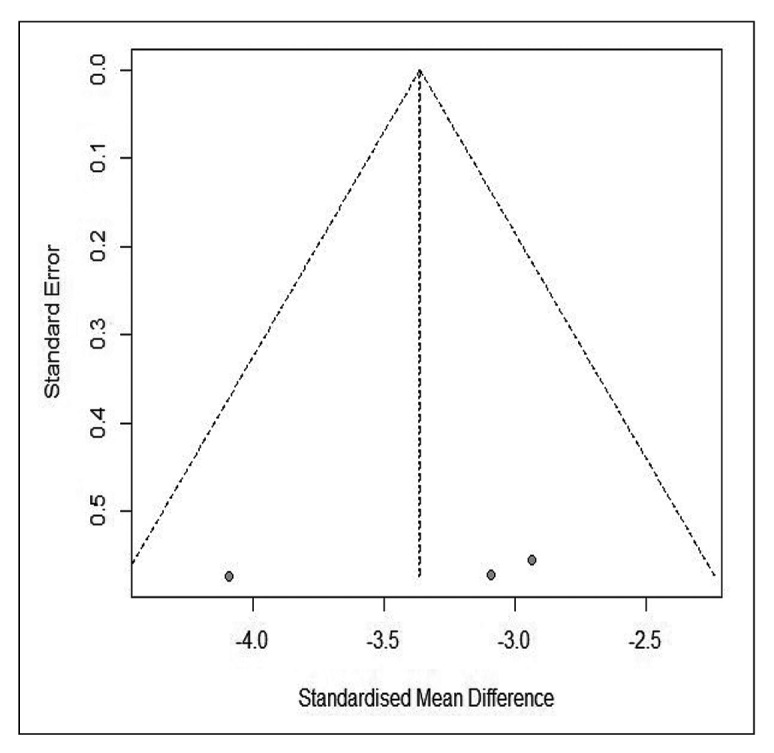
Funnel plot of controlled studies reporting prevalence of fasting normogastria in children.

**SUPPLEMENTAL FIGURE 2.**
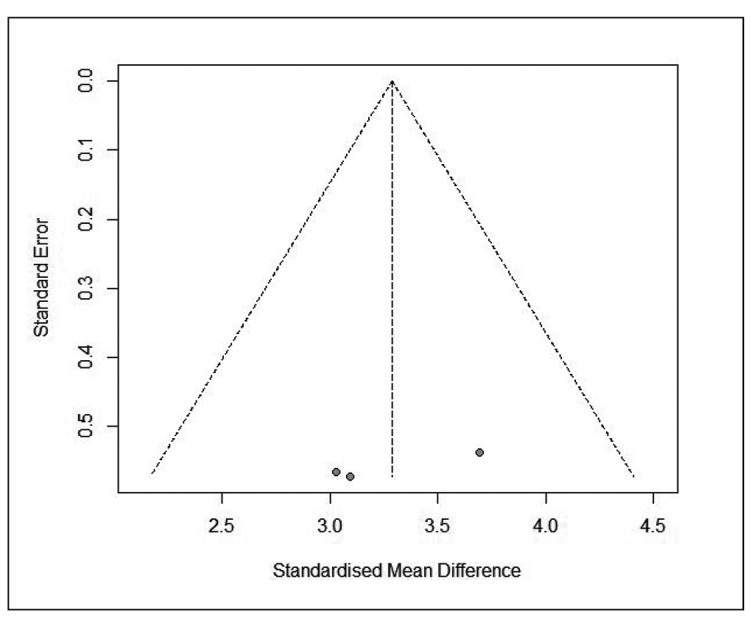
Funnel plot of controlled studies reporting prevalence of fasting tachygastria in children.

**SUPPLEMENTAL FIGURE 3.**
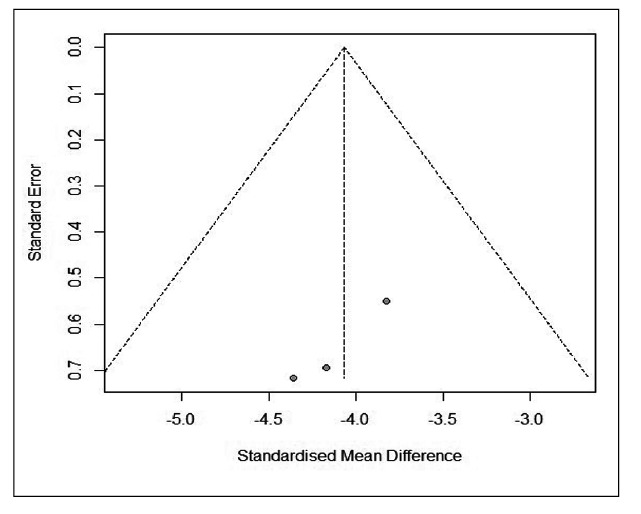
Funnel plot of controlled studies reporting power ratio in children.

Funnel plots were constructed on the pooled analyses that compared the electrogastrogram results between the gastroparetic and control groups (SUPPLEMENTARY FIGURES 1, 2, AND 3). Asymmetry was evident in all funnel plots; however, Egger’s test could not be evaluated because the number of studies was too small (less than 10 studies) to test the small-study effect. We evaluated the Vevea and Hedges weight function model for publication bias assessment of the combined analysis results of fasting normogastria, fasting tachygastria, and the power ratio. As shown in Table 2, all *P* values were greater than 0.1; thus, there is evidence that there was no publication bias.

**TABLE 2.** Results of the Vevea and Hedges Weight Function Model.

	Estimated model not adjusted	Adjusted model estimate	***P*-value of the likelihood ratio test**
Fasting normogastria	-3.359	-3.359	1
Fasting tachygastria	3.285	3.285	0.99128
Power ratio	-4.071	-4.071	1

## DISCUSSION

We conducted a systematic review and meta-analysis to evaluate gastric myoelectrical activity by assessing gastric slow waves using electrogastrography in children with gastroparesis. From the systematic search carried out in five databases, we were able to identify four articles that met our inclusion criteria, presenting the limitation of not being able to include articles in languages other than English and Spanish, since most did not have access to the full text.

Our first outcome evaluated was the percentage of records in which the dominant power corresponded to normogastria, tachygastria, and bradygastria. Studies with children have found a lower percentage of fasting normogastria in children with gastroparesis. However, only the study by Cucchiara et al. reported that the percentage of fasting normogastria in control children was significantly higher than that in controls when compared with gastroparetic children^30^. Carson et al. evaluated nausea and vomiting syndromes in adults and found prevalence rates of 50.45% and 52.8% in fasting and postprandial normogastria, respectively^33^. Our results for the percentage of fasting normogastria in gastroparetic children were lower than the normal value in healthy humans, which is greater than 70%^14^. The percentage of post-stimulus normogastria in gastroparetic children was in the normal range, considered to be greater than 70%^14^.

Our percentage of fasting bradygastria in gastroparetic children was 14.6% (95%CI: 0.6-41.9%), which was higher than the percentage reported in control children in a single study (3.8%±2.7%)^30^. We found only one study that reported the percentage of post-stimulus bradygastria in gastroparetic children, with 15.9%±13%, without a control group. When comparing our percentages with those of Carson et al., we found lower values in both fasting (34.1%) and post-stimulus (24.56%) conditions^33^. Our percentage of fasting tachygastria in gastroparetic children was 18.7% (95%CI: 9.8-29.7%), which was higher than the percentage reported in control children in a single study (8.4%±6.6%)^30^. Only one study reported that the percentage of post-stimulus tachygastria in gastroparetic children was 3.1%±5.1% without a control group. Comparing our results with those of Carson et al., they were similar in fasting (20.29%), but lower in post-stimulus (21.34%)^33^.

Only one pediatric study reported DFIC values in fasting (0.256) and post-stimulus gastroparetic patients (0.186)^29^. Although the clinical relevance of this parameter in electrogastrography has not yet been established, DFIC was introduced to specify the stability of the DF of the electrogastrogram. That is, while the percentage of normal gastric slow waves specifies the regularity of the gastric slow wave, DFIC reflects subtle changes in the gastric slow wave, showing subtle variations in the dominant frequency within the specified range^14^.

According to our meta-analysis, the power ratio was significantly lower in gastroparetic patients compared to controls. Our results were similar to those reported by Carson et al., in which there was a moderate decrease in the increase in post-stimulus power in gastroparetic patients compared to controls (SMD: -0.62, 95%CI: -2.10 to 0.86)^33^. Electrogastrogram dominant power was proportional to the regularity and amplitude of the gastric slow wave, increasing when the gastric slow wave became more regular or when there was an increase in the amplitude of the gastric slow wave. However, the absolute value of the dominant power may not be significant, as it can be affected by many factors such as electrode position, skin preparation, and abdominal wall thickness^14^. The power ratio is a parameter associated with alterations in gastric contractions. A ratio >1 reflects an increase in gastric contractility due to the intervention, whereas a ratio <1 reflects a decrease in gastric contractility^14^. In our study, it was shown that the power ratio in gastroparetics was less than one, while controls showed an increase 3.4 times greater than in gastroparetics, indicating a possible alteration in gastric contractibility and/or distension in these patients.

Gastric biopsies in patients with gastroparesis have reported alterations in CD3+ cells^32^. Studies have described other inflammatory changes, such as increased macrophages, lymphocyte infiltrates, serum tumor necrosis factor α (88%), and serum interleukin-6 (91%)^2^ ^-^ ^6^. We did not find pediatric studies that evaluated ICC status in gastric biopsies. Studies have described the loss of ICC^2^ ^-^ ^7^. A decrease in ICC number has been associated with electrogastrogram abnormalities^7^. These cells have pacemaker activity, generated by those located in the myenteric region (ICC-MY), and binding to other cells allows slow wave generation and subsequent smooth muscle contraction. Two types of gastric electrical waves have been described: the first are potential pacemaker signals, which precede the second type, known as slow waves. The former is generated by the ICC-MY and the latter by smooth muscle cells^34^ ^-^ ^36^. In the absence of ICCs, there are alterations in electrical rhythmicity, with a failure to show any slow-wave action potential^37^. Another type of ICC is intramuscular ICC (ICC-IM), which generates the secondary regenerative component of a slow wave, increasing the depolarization that arises from ICC-MY. The lack of ICC-IM results in the absence of a secondary component in the depolarization waves of smooth muscle cells^38^.

Heterogeneity was evaluated using the I^2^ and PI values. The I^2^ values varied according to the variables used in the meta-analysis. In meta-analyses that summarized proportions (percentage of duration of registration in normogastria, bradygastria, and tachygastria), 50% had I^2^ greater than 95%, one in 0%, and another in 75%. In meta-analyses summarizing the means (power ratio), only one study could be performed with children, with an I^2^ of 53%. Meta-analyses comparing electrogastrogram measurements in gastroparetics with controls using the SMD, the studies in children, all I^2^ were less than 20%. When I^2^ was calculated in prevalence meta-analyses, the estimated I^2^s were found to be high, with a median I^2^ of 96.9% (interquartile range: 90.5-98.7)^39^. A meta-analysis evaluating other data types found a median I^2^ of 21.1% (interquartile range: 0.0-49.7), with 5.6% of the analyses having an I^2^ ≥75%^40^. We found an I^2^ of 0% in our meta-analysis for post-stimulus normogastria in gastroparetic children, possibly secondary to the fact that it only included two studies^29^ ^,^ ^32^. One study demonstrated that the prevalence meta-analyses, including fewer studies, had a low I^2^ ^39^. In addition, it has been erroneously concluded that low I^2^ values indicate homogeneity, whereas high I^2^ values indicate heterogeneity^11^.

As previously mentioned, we also calculated PI. This is a dispersion index based on the standard deviation, which indicates how much the effects vary between populations and is not related to the number of studies in the analysis^27^. Our prevalence meta-analysis (normogastria in fasting gastroparetic children) had a PI similar to the CI of the summary estimator, all presenting I^2^ ≥75%. The meta-analysis of the power ratio showed a PI similar to the CI of the summary estimator, with an I^2^ value of 53%. A possible explanation for this wide PI is that they occur more frequently in meta-analyses with continuous results^27^. Our meta-analysis included a few studies with asymmetry in the visual inspection of funnel plots. Prediction intervals are strongly based on the assumption of a normal distribution of effects across studies and can be very problematic when the number of studies is small, in which case they may appear falsely broad or narrow. Therefore, their use is recommended when the number of studies is reasonable (e.g., more than ten) and there is no clear asymmetry in the funnel plot^11^.

This study has some limitations. First, it was not possible to review all studies found because of the limitations in full-text access. Second, few pediatric studies have analyzed measures such as DF. Third, considerable heterogeneity in most meta-analyses influences variation between studies as random-effects meta-analyses weigh studies almost equally, regardless of sample size, resulting in a meta-analytic summary close to the arithmetic mean. Fourth, owing to the characteristics of the low-resolution electrogastrogram, we could not evaluate the propagation velocity of slow waves. Fifth, there is an absence of information on how to interpret the DFIC. Further studies in children are required to establish a protocol that includes recording times, type of electrodes, patient preparation, placement of the electrodes, reference values of electrogastrogram parameters, and stimuli for the complete evaluation of the patient. In this study, we concluded that fasting gastroparetic children had a lower percentage of normogastria and a higher percentage of tachygastria. Children with gastroparesis have a smaller increase in post-stimulus power, reflecting possible alterations in gastric contraction and/or distension.
